# Supplementary material for: Keyhole fluctuation and pore formation mechanisms during laser powder bed fusion additive manufacturing
Source: Nat Commun. 2022 Mar 4;13:1170. doi: 10.1038/s41467-022-28694-x (PMC8897468; doi:10.1038/s41467-022-28694-x)
Supplement: Supplementary file 1 — Supplementary Information [file 41467_2022_28694_MOESM1_ESM.pdf]

Supplementary Information

Keyhole fluctuation and pore formation mechanisms during laser powder bed fusion additive manufacturing

Yuze Huang[1,2*], Tristan G. Fleming[3], Samuel J. Clark[1,2,4], Sebastian Marussi[1,2], Kamel Fezzaa[4], Jeyan Thiyagalingam[5], Chu Lun Alex Leung[1,2*], Peter D. Lee[1,2*]

[1]UCL Mechanical Engineering, University College London, WC1E 7JE, UK

[2]Research Complex at Harwell, Harwell Campus, Didcot, OX11 0FA, UK

[3]Department of Physics, Queen's University, Kingston, Ontario, K7L 3N6, Canada

[4]X-ray Science Division, Argonne National Laboratory, Lemont, IL 60439, US

[5]Science and Technology Facilities Council, Harwell Campus, Didcot, OX11 0FA, UK

*emails: yuze.huang@ucl.ac.uk, alex.leung@ucl.ac.uk and peter.lee@ucl.ac.uk

This PDF file includes:

Supplementary Figs. 1-13

Supplementary Discussions 1-2

Supplementary Tables 1-4

Supplementary References

Supplementary Figures

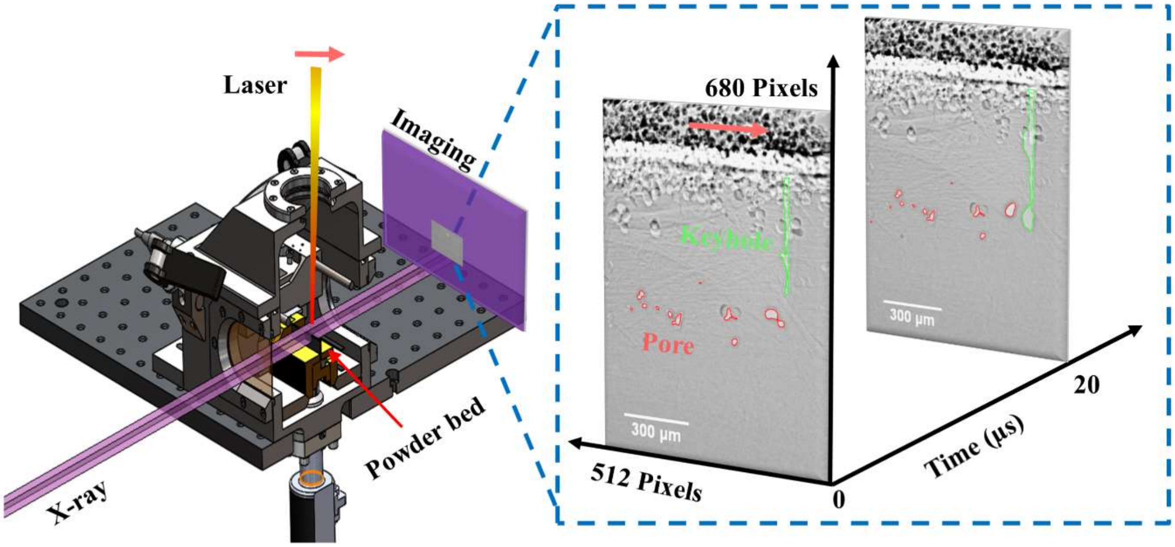

Supplementary Fig. 1. Schematic of the in situ synchrotron X-ray imaging experimental setup showing laser beam interacts with the powder bed whilst capturing time-series radiographs with a field of view of 512 pixels × 680 pixels (1 mm × 1.3 mm).

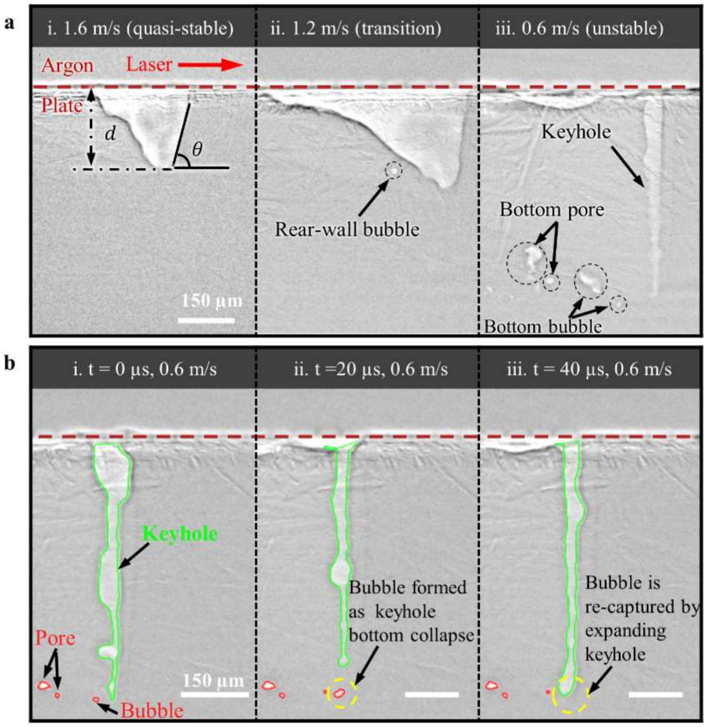

Supplementary Fig. 2. **a** Keyhole morphology variation with different types of bubble formation during laser melting of a bare aluminium plate at a laser power of 500W. d and θ represent the keyhole depth and front keyhole wall (FKW) angle, respectively. **b** Time series radiographs showing bubble is recaptured by an expanding keyhole.

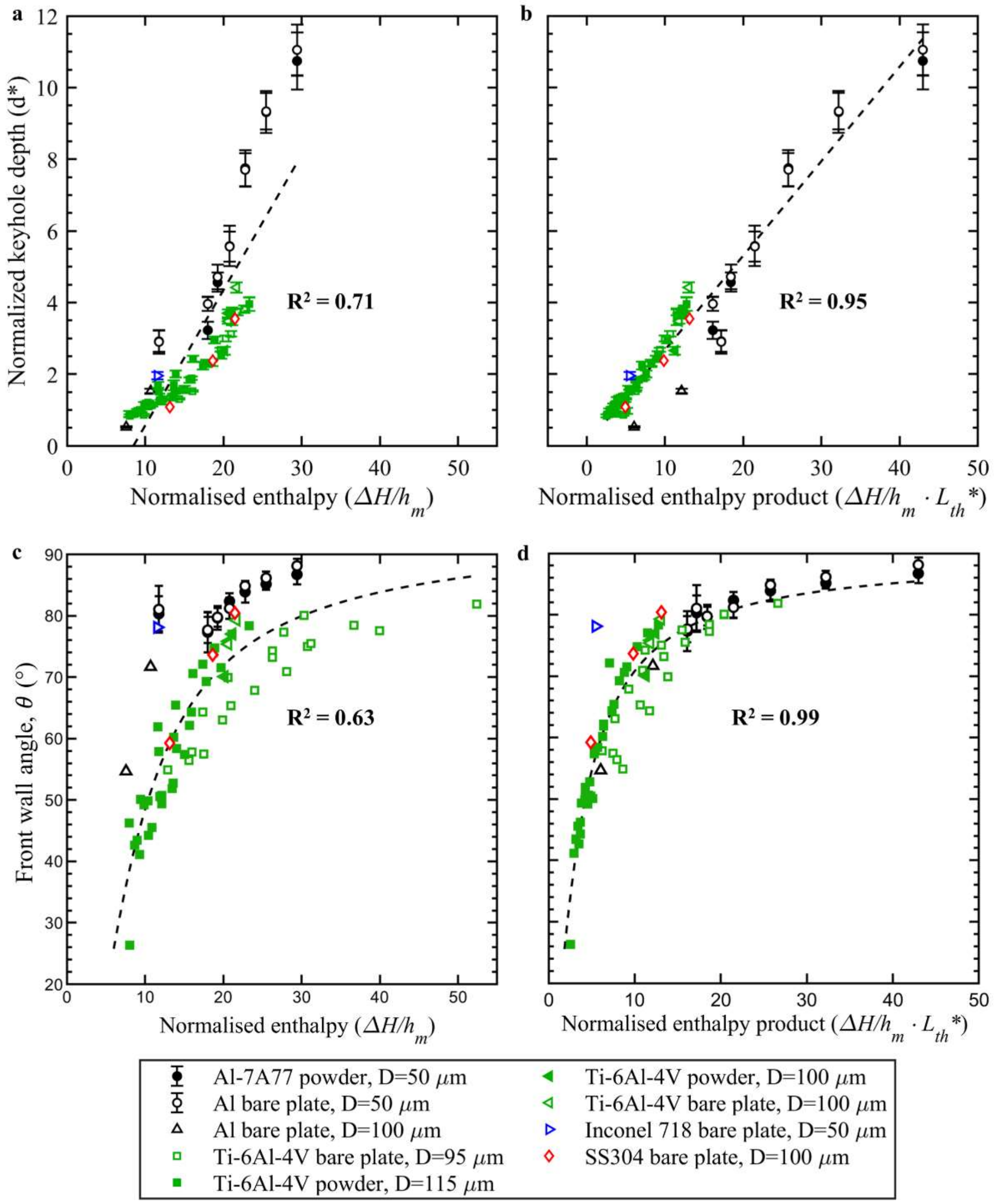

Supplementary Fig. 3. Comparing the scaling relations of keyhole depth (normalised by the laser spot size) and front keyhole wall (FKW) angle with the normalised enthalpy and normalised enthalpy product. The normalised keyhole depth as a function of **a** normalised enthalpy and **b** normalised enthalpy product for 9 datasets with 4 different materials. FKW angle as a function of normalised enthalpy **c** and normalised enthalpy product **d** for 9 datasets with 4 different materials. Note, **d** has been shown in Fig 2b, which is used here for comparing with **c**. The data points for LPBF of Al7A77 powder and bare aluminium plate were measured in this study, the Ti-6Al-4V data points were cited from Cunningham et al.[1] (Figs.4, S5 and S7) and Zhao et al.[2] (Movie S1 -Movie S5) with permission by AAAS. Data for LPBF with Inconel 718, SS 304 and aluminium bare plate were cited from Kouraytem et al.[3], Parab et al.[4], and Hojjatzadeh et al.[5], respectively. Error bars represent standard deviation.

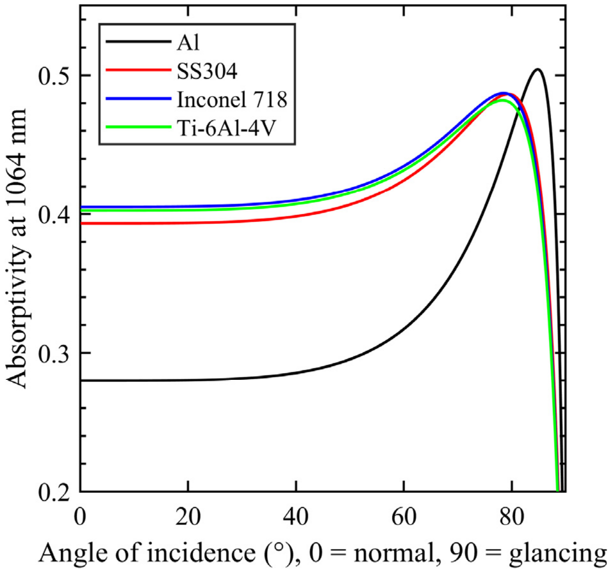

Supplementary Fig. 4. The laser absorptivity (or the Fresnel absorption) at the surface of molten Al, SS304, Inconel 718, and Ti-6Al-4V with respect to different angles of incidence. The Brewster angles are ~ 84.9°, 79.3°, 78.5° and 78.3° for Al, SS304, Inconel 718 and Ti-6Al-4V, respectively. The associated physical property values for the absorptivity calculation are shown in Supplementary Table 3 (material density) and Supplementary Table 4.

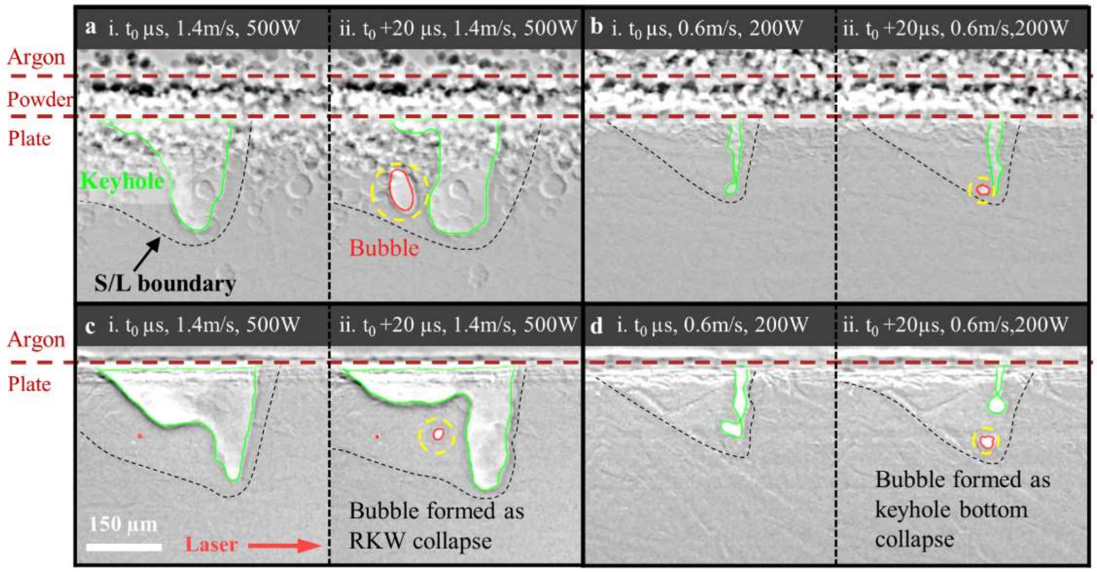

Supplementary Fig. 5. Keyhole morphology variations with different kinds of bubble formation at **a**, **c** high-power-velocity (high-PV) and **b**, **d** low-power-velocity (low-PV) while keeping similar area energy density (AED, $AED = 7\ MJ \cdot m^{-2}$) during LPBF with and without Al7A77 powder. t_0 is the time of the captured frame before the bubble is being pinched off. The keyhole (green) and bubble (red) are tracked by the developed algorithm depicted in Method. The bubbles of interest are highlighted with circular yellow-dash-line. RKW represents rear keyhole wall.

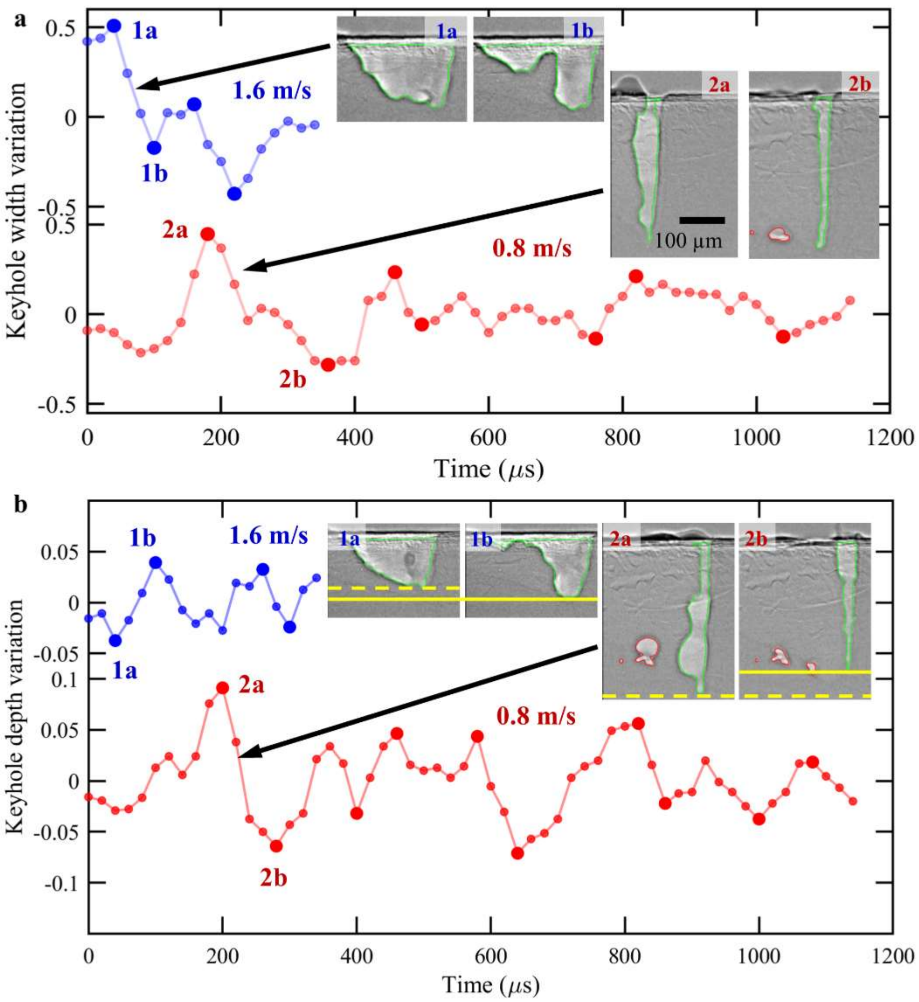

Supplementary Fig. 6. Keyhole width **a** and keyhole depth **b** relative to the means. Laser melting of pure aluminium plate at scan velocity of 1.6 m/s (top, blue) and 0.8 m/s (bottom, red). Detected peaks/valleys are highlighted with marker size and example X-ray radiographs (1a, b; 2a, b). Laser power 500 W.

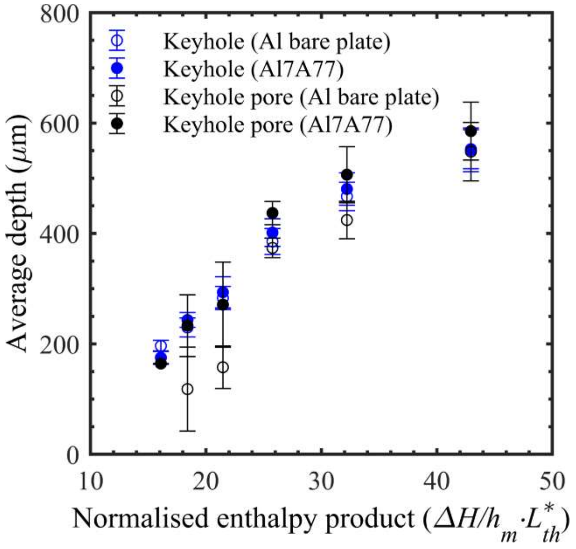

Supplementary Fig. 7. Compared the average keyhole depth with that of the average pore depth (distance from top of powder bed to the centre of pore) at different normalised enthalpy products. Error bars represent standard deviation.

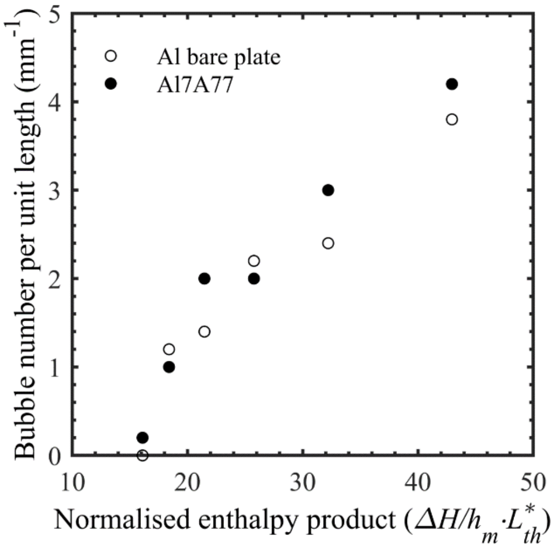

Supplementary Fig. 8. Compared the tracked bubble number from the in situ synchrotron X-ray images with and without powder at different normalised enthalpy products. Only the bubbles were induced by keyhole collapse were tracked here, excluding the bubbles formed by bubble splitting.

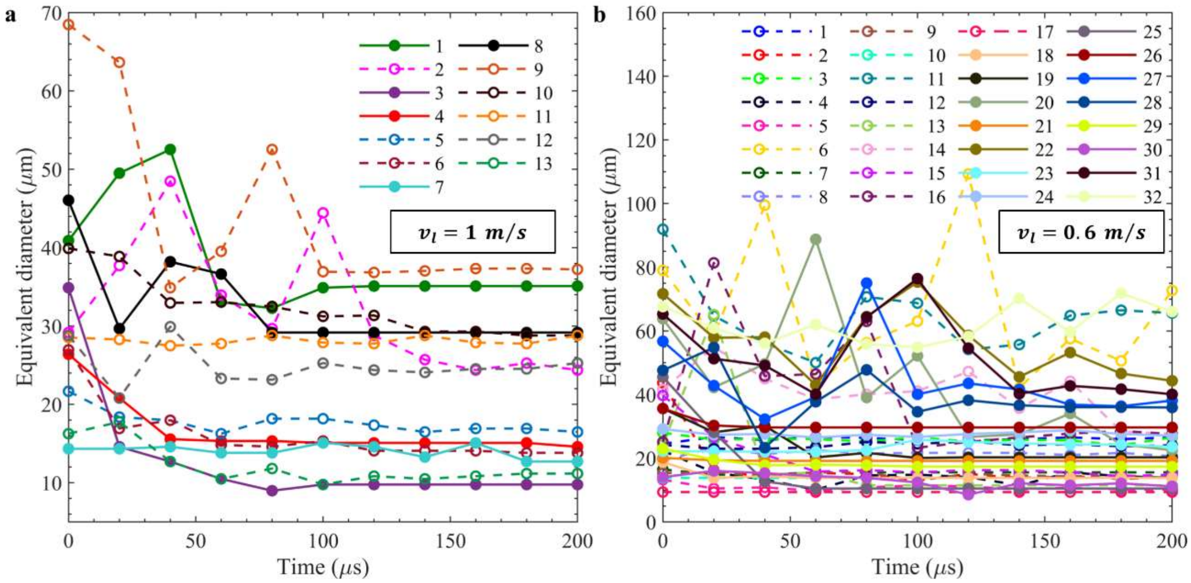

Supplementary Fig. 9. Equivalent diameter variations of all tracked keyhole bubbles during LPBF with (solid line) and without (dash line) Al7A77 powder. **a** Laser scan velocity of 1 m/s and laser power of 500 W. **b** Laser scan velocity of 0.6 m/s and laser power of 500 W. Note, identical graphs with error bars in Fig.3 and Supplementary Fig. 10. The equivalent diameter is calculated using $\sqrt{6A/\pi}$, where A is the bubble area measured from X-ray image (see details in Methods). The tracking algorithm sets the minimum number of frames that a bubble is identified as 6. The time 0 is set to the moment a bubble is first identified.

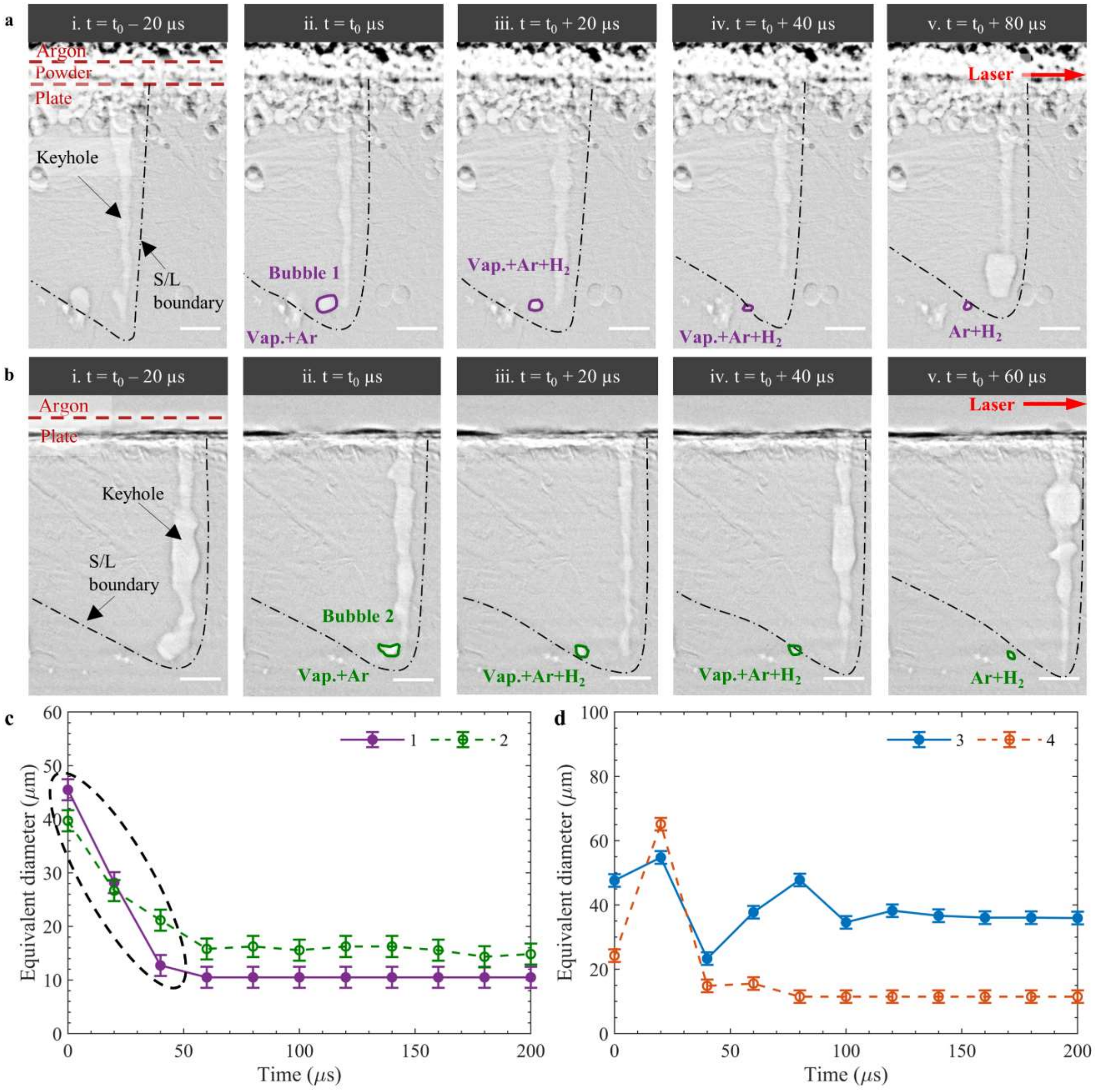

Supplementary Fig. 10. Keyhole bubble lifetime dynamics during LPBF. Laser scan velocity 0.6 m/s and laser power 500 W. **a** and **b** are radiographs with Al7A77 powder and bare aluminium plate, respectively. **c** and **d** show the equivalent diameter changes of some tracked bubbles in LPBF with (solid line) and without (dash line) Al7A77 powder. The equivalent diameter is calculated using $\sqrt{6A/\pi}$, where A is the bubble area measured from X-ray image (see details in Methods). Note the bubble size error is calculated as ± 2 pixels (*ca.* 4 μm), equivalent to the segmentation uncertainty. The total tracked bubble numbers are 15 and 17 for the powder and bare plate cases (see Supplementary Fig. 9b), respectively, using a criterion where the minimum number of frames that a bubble is identified is 6. The time t_0 is set to the moment a bubble is first identified (Note, t_0 is set as $t_0=0$ in **c** and **d**). The black dashed circles show stage (2) bubble shrink due to vapour condensation. The bubbles of interest shown in **a** and **b** are marked by purple and green colours, respectively, corresponding to same colours in **c**. Vap.: Vapour; Ar: Argon; H_2: Hydrogen. All scale bars correspond to 100 μm.

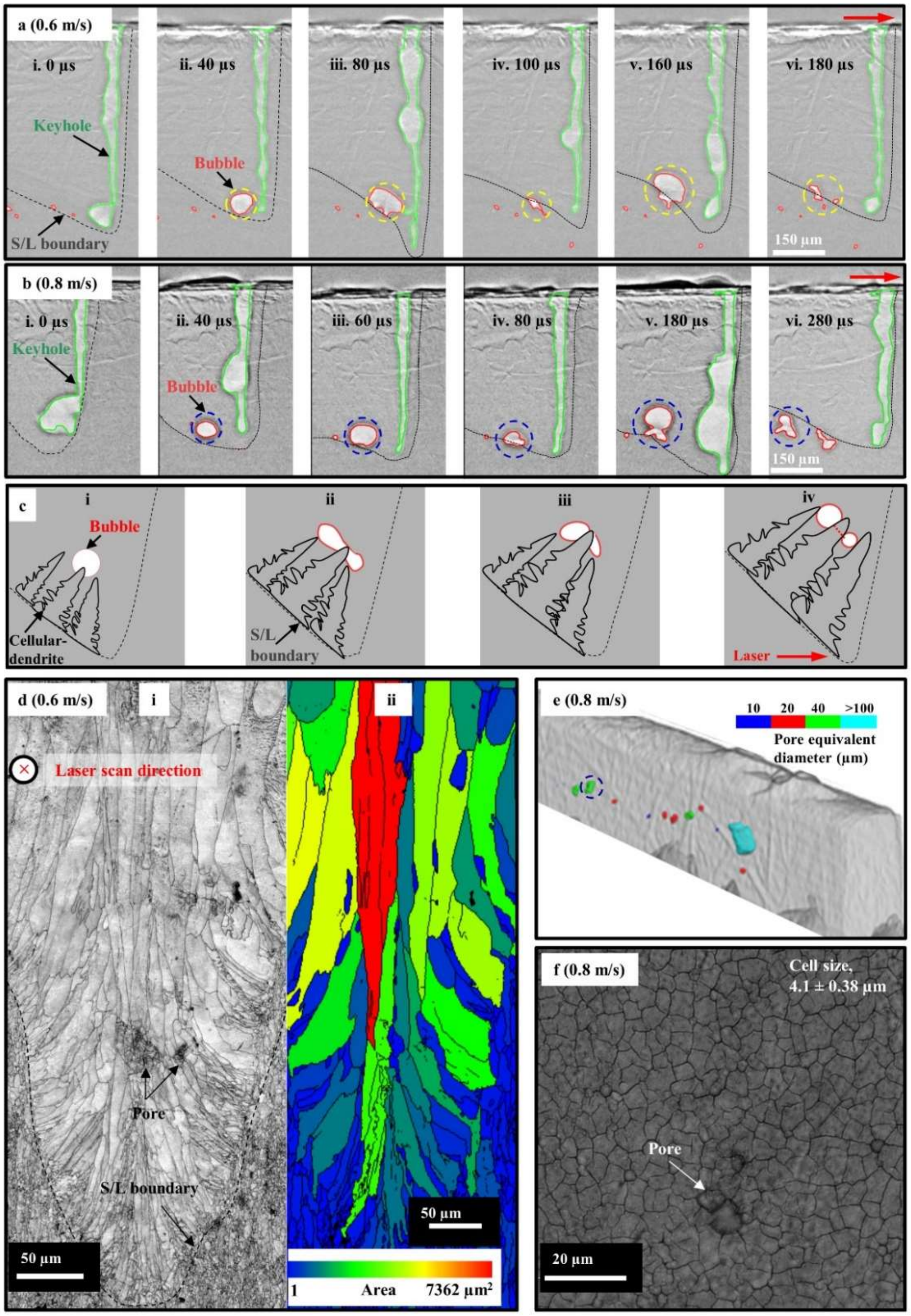

Supplementary Fig. 11. Bubble dynamics induced by the cellular-dendrite growth over the solid/liquid (S/L) boundary. **a**, **b** Bubble behaviour revealed by in situ synchrotron X-ray imaging under laser power of 500 W and laser scan velocities of 0.6 m/s and 0.8 m/s, respectively. **c** Illustration showing changes in bubble size due to cellular-dendritic growth. **d** SEM image of the 0.6 m/s sample showing the S/L boundary, pore, and grain structure, in which the grain area is measured by EBSD. (ii). **e** Micro-computed tomography of the track sample overlaid with pore equivalent diameter. **f** A micrograph of the 0.8 m/s sample captured by a microscope VHX-7000 (Keyence, Itasca, USA).

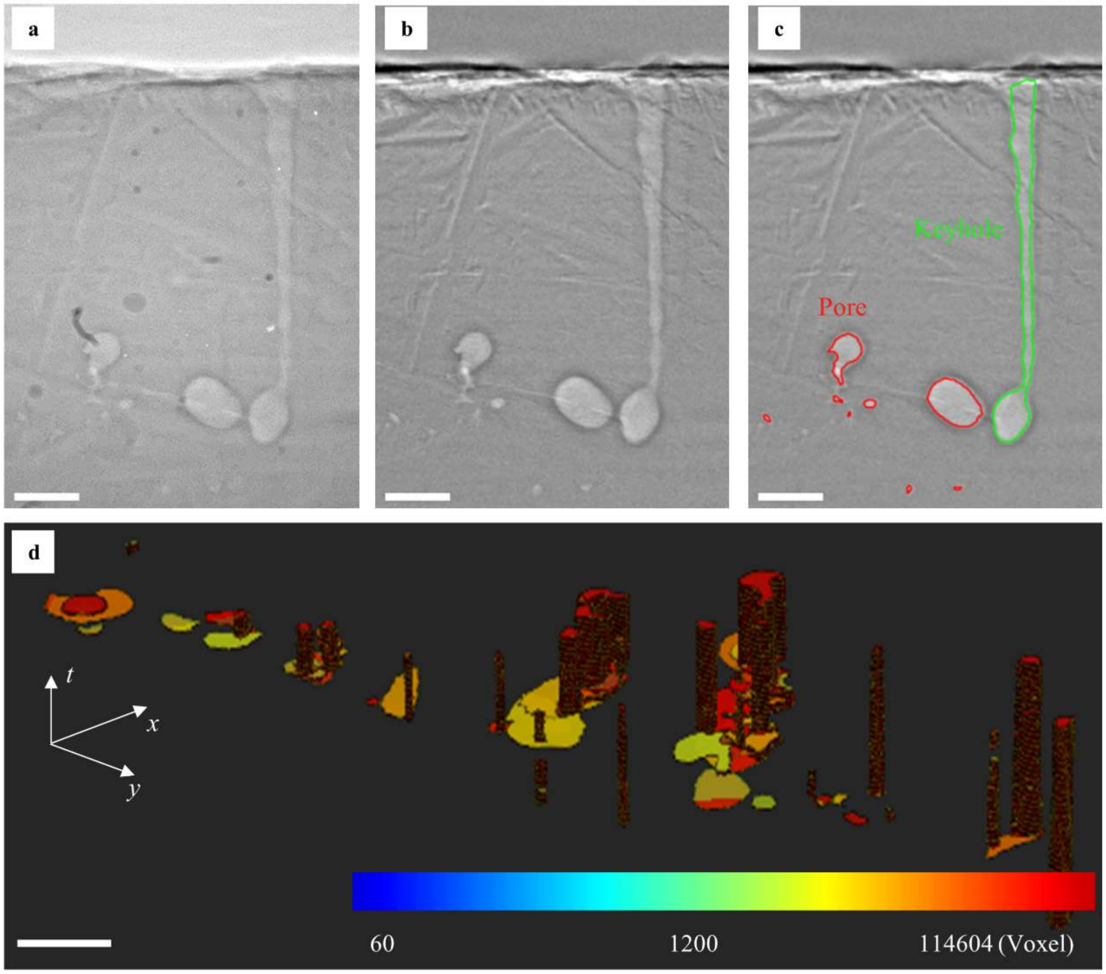

Supplementary Fig. 12. Image processing sequence for this study. **a** Original image. **b** Flat field correction, subtracting the offset background, followed by Gaussian filtering. **c** Feature segmentation. **d** Frame stack integration with voxel thresholding in time domain. Keyhole is marked by green boundary while pores are marked by red boundary. All white scale bars correspond to 100 μm.

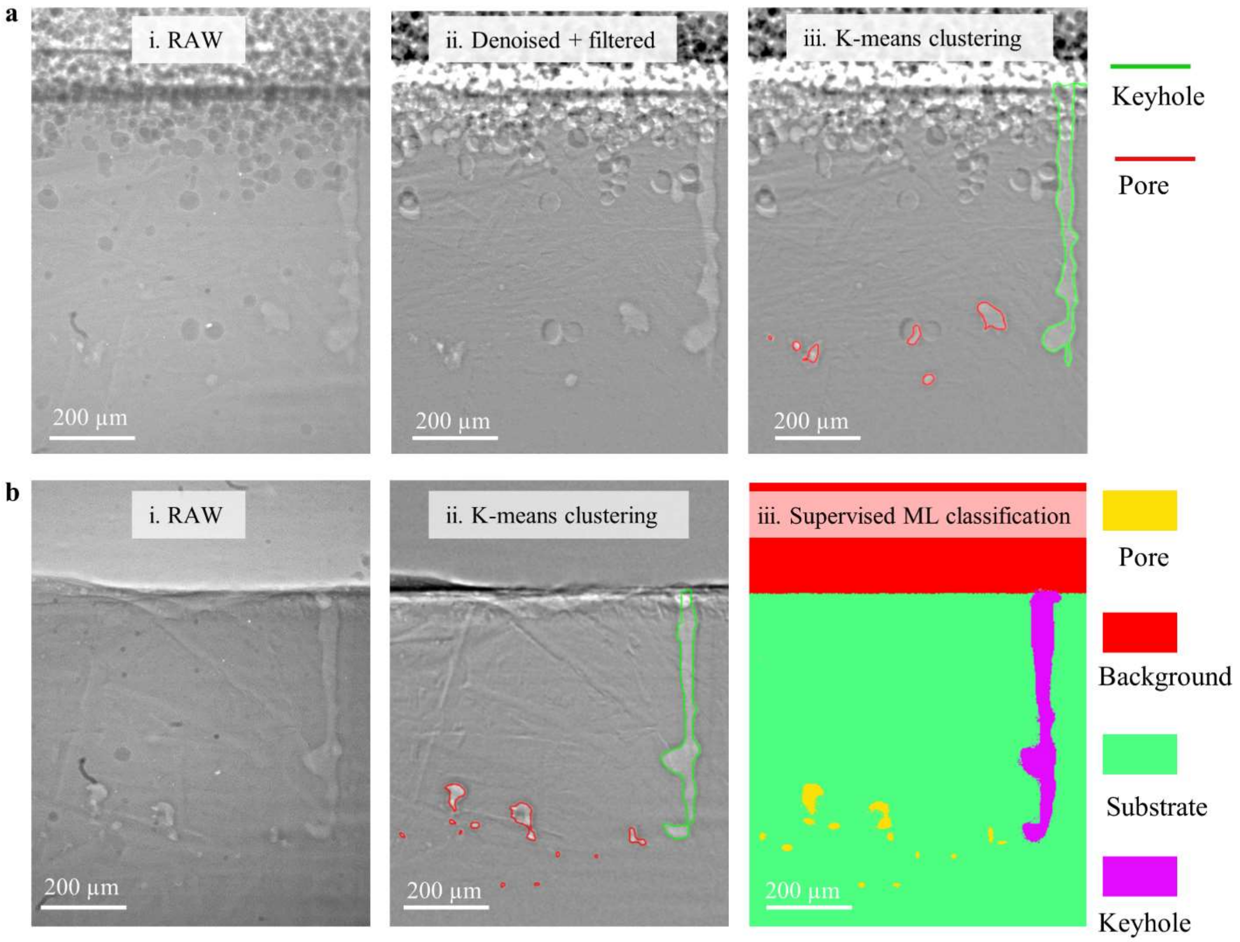

Supplementary Fig. 13. Analysis and feature extraction for X-ray synchrotron radiographs of LPBF. **a** Keyhole and keyhole pore extraction via K-means clustering for LPBF with Al7A77 powder. **b** Keyhole and keyhole pore extraction via K-means clustering and supervised machine learning (random decision forests) for laser melting without powder.

Supplementary Discussion 1

Hump formation on front keyhole wall (FKW)

Hump formation on FKW is driven by the dependence of laser absorptivity on angle of incidence[6–9], defined by the Fresnel curve for a given material (Supplementary Fig. 4), causing non-uniform laser absorption (Fresnel absorption) and temperature over FKW. Once the temperature of the local zone of FKW is high enough to induce intense evaporation and resulting recoil pressure force, a small hump may then be formed by the local vapor depression, which is usually originated at the top part of FKW. The resulting recoil pressure may then push the hump downward along FKW[10,11]. Meanwhile, new humps can occur repeatedly at the top of FKW. Based on our experimental observations (Supplementary Movies 1 –

12), we also found that the hump generation frequency increases with an increase of laser scan velocity while its magnitude decreases, being consistent with the prior reports[11,12].

Supplementary Discussion 2

The interaction of bubbles with solidification microstructure

As bubbles interact with the solid/liquid (S/L) boundary, bubbles can show sudden bursts of growth and shrinkage over the solidification front, presented in Supplementary Fig. 11a, b with different laser scan velocities. We hypothesise that this phenomenon is induced by the interaction of the rapidly growing microstructure and the bubble, which were also observed in the solidification of aluminium alloy during casting[13,14], illustrated in the Supplementary Fig. 11c. Due to the cellular-dendrite growth, the bubble shape may be squeezed in the observed plane of synchrotron X-ray imaging. There is a chance that the bubble is partially hided by the growing cellular structure, and only partial bubble can be clearly observed by using the synchrotron X-ray imaging. It was found that the final captured pore's morphology by in situ synchrotron X-ray (Supplementary Fig. 11b, 280μs) is more like a specific cross-section of the final 3D pore presented by the micro-computed tomography (μCT, shown in Supplementary Fig. 11e), which is partially hided by the cellular-dendrites as the bubble got wrapped around the dendrites. Note that we used similar locations (e.g., centroids) of the pores to correlate between in situ synchrotron X-ray and ex situ μCT, which were highlighted in purple-dash-circle lines in Supplementary Fig. 11e and Supplementary Fig. 11b, respectively.

To further check the microstructure, ex situ micrographs (Supplementary Fig. 11d, f) were also achieved over the transverse cross-section of the samples. As noticed from Supplementary Fig. 11d, f, grains with size around 30 ~ 100 μm grow perpendicular to the melt pool border but towards the melt pool centre. A closer look with higher magnification (Supplementary Fig. 11f) shows much finer cellular–dendritic solidification structure with a size of 4.1 ± 0.38 μm. The measured sizes of grain and cellular are consistent with prior reports of aluminium alloy in LPBF, recorded as ~ 50 μm and ~ 1μm for grain and cellular[15], respectively. Those ex situ micrographs (Supplementary Fig. 11d, f) confirm that the pore can

be engulfed by the cellular microstructure, in turn, the cellular size around the pore can cause disruption to cellular growth, serving as thermal insulation, decreasing the localised solidification rate and temperature gradient[16], and finally leading to different cellular sizes on different sides of the pore, shown in Supplementary Fig. 11f.

Supplementary Tables

Supplementary Table 1.Characteristic parameters of the built bubble growth model in Methods. Note, the laser absorptivity at the surface of molten Al was calculated with respect to the measured front keyhole wall angle (Supplementary Fig. 4). Laser power 500 W, laser spot size 100 um, ambient pressure $1.11 \times 10^5\ Pa$.

Bubble initial radius, r_{b0} (μm)	Scan velocity, v_l ($m \cdot s^{-1}$)	Absorptivity of molten Al	Bubble surface temperature, T_{bs} (K)	Recoil pressure, p_{recoil} ($10^5\ Pa$)	Pressure-driven bubble growth time, t_1(μs)
18.4	0.8	0.5	3463	23	5.4
21.8	1.2	0.45	1488	6	3.4

Supplementary Table 2. Material composition of the Al7A77 powder[17].

Aluminium	Chromium	Copper	Magnesium	Zinc	Zirconium	Trace Elements
Balance	< 0.1 %	1.1 - 2.1%	1.8 - 2.9%	4.5 - 6.1%	0.5 - 2.8%	< 0.15%

Supplementary Table 3. Thermophysical properties for pure aluminium, Ti-6Al-4V, SS304 and Inconel 718 at the liquidus temperature[18]. Absorptivity values are averages over reported values for an unpolished, oxidized flat surface (commercial-grade) at room temperature, as well as elevated temperatures[19–22] for laser beam with 1μm wavelength.

Material	Density ($kg \cdot m^{-3}$)	Liquidus temperature (K)	Heat capacity ($J \cdot kg^{-1} \cdot K^{-1}$)	Thermal diffusivity ($10^{-6}\ m^2 \cdot s^{-1}$)	Absorptivity	Melting enthalpy ($10^9\ J \cdot m^{-3}$)
Aluminium	2385	933.5	1180	32	0.15	2.627
Ti-6Al-4V	3920	1923	831	8.6	0.45	6.264
SS304	6900	1727	800	5.6	0.40	9.533
Inconel 718	7400	1609	720	5.6	0.40	8.573

Supplementary Table 4. Physical property values for the studied materials, fed into the Fresnel/Drude model for absorptivity of a metal surface with varying angle of incidence (Elaborated in the reference of Mahrle and Beyer[23]). The property values of aluminium, Ti-6Al-4V, SS304 and Inconel 718 were cited from references[24–27].

Material	Valence electrons per atom	Electric resistivity (10^{-8} Ω·m)	Atomic mass (10^{-3} kg·mol^{-1})
Aluminium	3	25	27
Ti-6Al-4V	2	176	47
SS304	4	150	56
Inconel 718	2	169	57

Supplementary References

1. Cunningham, R. *et al.* Keyhole threshold and morphology in laser melting revealed by ultrahigh-speed x-ray imaging. *Science (80-.).* **363**, 849–852 (2019).

2. Zhao, C. *et al.* Critical instability at moving keyhole tip generates porosity in laser melting. *Science (80-.).* **370**, 1080–1086 (2020).

3. Kouraytem, N. *et al.* Effect of Laser-Matter Interaction on Molten Pool Flow and Keyhole Dynamics. *Phys. Rev. Appl.* **11**, 064054 (2019).

4. Parab, N. D. *et al.* Ultrafast X-ray imaging of laser–metal additive manufacturing processes. *J. Synchrotron Radiat.* **25**, 1467–1477 (2018).

5. Hojjatzadeh, S. M. H. *et al.* Direct observation of pore formation mechanisms during LPBF additive manufacturing process and high energy density laser welding. *Int. J. Mach. Tools Manuf.* **153**, (2020).

6. Tan, W. & Shin, Y. C. Analysis of multi-phase interaction and its effects on keyhole dynamics with a multi-physics numerical model. *J. Phys. D. Appl. Phys.* **47**, (2014).

7. Kaplan, A. F. H. & Matti, R. S. Absorption peaks depending on topology of the keyhole front and wavelength. *J. Laser Appl.* **27**, S29012 (2015).

8. Kaplan, A. F. H. Fresnel absorption of 1 μm- and 10 μm-laser beams at the keyhole wall during laser beam welding: Comparison between smooth and wavy surfaces. *Appl. Surf. Sci.* **258**, 3354–3363 (2012).

9. Eriksson, I. New high-speed photography technique for observation of fluid flow in laser welding. *Opt. Eng.* **49**, 100503 (2010).

10. Zhao, C. *et al.* Bulk-explosion-induced metal spattering during laser processing. *APS* **9**, (2019).

11. Courtois, M., Carin, M., Le Masson, P., Gaied, S. & Balabane, M. A complete model of keyhole and melt pool dynamics to analyze instabilities and collapse during laser welding. *J. Laser Appl.* **26**, 042001 (2014).

12. Matsunawa, A., Kim, J., … S. K.-… on A. of & 1996, undefined. Experimental and theoretical studies on keyhole dynamics in laser welding. *lia.scitation.org* 58 (1996) doi:10.2351/1.5059026.

13. Lee, P. D., Wang, J. & Atwood, R. C. Microporosity Formation during the Solidification of Aluminum-Copper Alloys. *JOM* 5 (2009).

14. Lee, P. D. & Hunt, J. D. Model of the interaction of porosity and the developing microstructure. in *Modeling of Casting, Welding and Advanced Solidification Processes* 585–592 (1995).

15. Thijs, L., Kempen, K., Kruth, J. P. & Van Humbeeck, J. Fine-structured aluminium products with controllable texture by selective laser melting of pre-alloyed AlSi10Mg powder. *Acta Mater.* **61**, 1809–1819 (2013).

16. Pham, M. S., Dovgyy, B., Hooper, P. A., Gourlay, C. M. & Piglione, A. The role of side-branching in microstructure development in laser powder-bed fusion. *Nat. Commun.* **11**, (2020).

17. HRL Laboratories. Aluminium 7A77 Data Sheet. https://www.hrl.com/products-services/materials/_assets/7A77-data-sheet.pdf.

18. Mills, K. C. *Thermophysical Properties of Selected Commercial Alloys*. (2011).

19. Rubenchik, A. M. *et al.* Temperature-dependent 780-nm laser absorption by engineering grade aluminum, titanium, and steel alloy surfaces. *Opt. Eng.* **53**, 122506 (2014).

20. Boley, C. D., Mitchell, S. C., Rubenchik, A. M. & Wu, S. S. Q. Metal powder absorptivity: modeling and experiment. *Appl. Opt.* **55**, 6496 (2016).

21. Rubenchik, A. *et al.* Direct measurements of temperature-dependent laser absorptivity of metal powders. *Appl. Opt.* **54**, 7230 (2015).

22. Trapp, J., Rubenchik, A. M., Guss, G. & Matthews, M. J. In situ absorptivity measurements of metallic powders during laser powder-bed fusion additive manufacturing. *Appl. Mater. Today* **9**, 341–349 (2017).

23. Mahrle, A. & Beyer, E. Theoretical aspects of fibre laser cutting. *J. Phys. D Appl.* **42**, 9 (2009).

24. Pawel, R. E. & Williams, R. K. *Survey of physical property data for several alloys*. (1985).

25. Milck, J. T. *Electrical resistivity data and bibliography on titanium and titanium alloys*. (1970).

26. Ho, C. Y. & Chu, T. K. *Electrical resistivity and thermal conductivity of nine selected AISI S*. (1977).

27. Brandt, R. & Neuer, G. Electrical Resistivity and Thermal Conductivity of Pure Aluminum and Aluminum Alloys up to and above the Melting Temperature. *Int. J. Thermophys.* **28**, 1429–1446 (2007).
