# Supplementary material for: Keyhole fluctuation and pore formation mechanisms during laser powder bed fusion additive manufacturing
Source: Nat Commun. 2022 Mar 4;13:1170. doi: 10.1038/s41467-022-28694-x (PMC8897468; doi:10.1038/s41467-022-28694-x)
Supplement: Supplementary file 3 — Description of Additional Supplementary Files [file 41467_2022_28694_MOESM3_ESM.pdf]

Description of Additional Supplementary Files

File Name: Supplementary Movie 1
Description: Dynamics of keyhole and keyhole bubbles during LPBF with 30 µm thick Al7A77 powder layer in the denoised synchrotron X-ray radiograph. The camera frequency rate is 50 kHz. The horizontal and vertical dimensions of the images are 1 mm and 1.33 mm, respectively. Laser power is 500 W, scan velocity is 0.6 m/s.

File Name: Supplementary Movie 2
Description: Segmented keyhole (green colour) and keyhole bubble (red colour) during LPBF with 30 µm thick Al7A77 powder layer in the denoised synchrotron X-ray radiograph. Laser power is 500 W, scan velocity is 0.6 m/s.

File Name: Supplementary Movie 3
Description: Dynamics of keyhole and keyhole bubbles during laser melting with bare aluminium plate in the denoised synchrotron X-ray radiograph. The camera frequency rate is 50 kHz. The horizontal and vertical dimensions of the images are 1 mm and 1.33 mm, respectively. Laser power is 500 W, scan velocity is 0.6 m/s.

File Name: Supplementary Movie 4
Description: Segmented keyhole (green colour) and keyhole bubble (red colour) during laser melting with bare aluminium plate in the denoised synchrotron X-ray radiograph. Laser power is 500 W, scan velocity is 0.6 m/s.

File Name: Supplementary Movie 5
Description: Dynamics of keyhole and keyhole bubbles during laser melting with bare aluminium plate in the denoised synchrotron X-ray radiograph. The camera frequency rate is 50 kHz. The horizontal and vertical dimensions of the images are 1 mm and 1.33 mm, respectively. Laser power is 500 W, scan velocity is 0.8 m/s.

File Name: Supplementary Movie 6
Description: Segmented keyhole (green colour) and keyhole bubble (red colour) during laser melting with bare aluminium plate in the denoised synchrotron X-ray radiograph. Laser power is 500 W, scan velocity is 0.8 m/s.

File Name: Supplementary Movie 7
Description: Dynamics of keyhole and keyhole bubbles during LPBF with 30 µm thick Al7A77 powder layer in the denoised synchrotron X-ray radiograph. The camera frequency rate is 50 kHz. The horizontal and vertical dimensions of the images are 1 mm and 1.33 mm, respectively. Laser power is 500 W, scan velocity is 1 m/s.

File Name: Supplementary Movie 8
Description: Segmented keyhole (green colour) and keyhole bubble (red colour) during LPBF with 30 µm thick Al7A77 powder layer in the denoised synchrotron X-ray radiograph. Laser power is 500 W, scan velocity is 1 m/s.

File Name: Supplementary Movie 9
Description: Dynamics of keyhole and keyhole bubbles during LPBF with 30 µm thick Al7A77 powder layer in the denoised synchrotron X-ray radiograph. The camera frequency rate is 50 kHz. The horizontal and vertical dimensions of the images are 1 mm and 1.33 mm, respectively. Laser power is 500 W, scan velocity is 1.2 m/s.

File Name: Supplementary Movie 10
Description: Dynamics of keyhole and keyhole bubbles during laser melting with bare aluminium plate in the denoised synchrotron X-ray radiograph. The camera frequency rate is 50 kHz. The horizontal and vertical dimensions of the images are 1 mm and 1.33 mm, respectively. Laser power is 500 W, scan velocity is 1.2 m/s.

File Name: Supplementary Movie 11
Description: Segmented keyhole (green colour) and keyhole bubble (red colour) during laser melting with bare aluminium plate in the denoised synchrotron X-ray radiograph. Laser power is 500 W, scan velocity is 1.2 m/s.

File Name: Supplementary Movie 12
Description: Dynamics of keyhole and keyhole bubbles during LPBF with 30 µm thick Al7A77 powder layer in the denoised synchrotron X-ray radiograph. The camera frequency rate is 50 kHz. The horizontal and vertical dimensions of the images are 1 mm and 1.33 mm, respectively. Laser power is 500 W, scan velocity is 1.6 m/s.
